# Effect of Water and Carbon Dioxide on the Performance of Basolite Metal–Organic Frameworks for Methane Adsorption

**DOI:** 10.1021/acs.energyfuels.3c02393

**Published:** 2023-09-27

**Authors:** David Ursueguía, Eva Díaz, Salvador Ordóñez

**Affiliations:** Catalysis, Reactors and Control Research Group (CRC), Department of Chemical and Environmental Engineering, University of Oviedo, Julián Clavería s/n, 33006 Oviedo, Spain

## Abstract

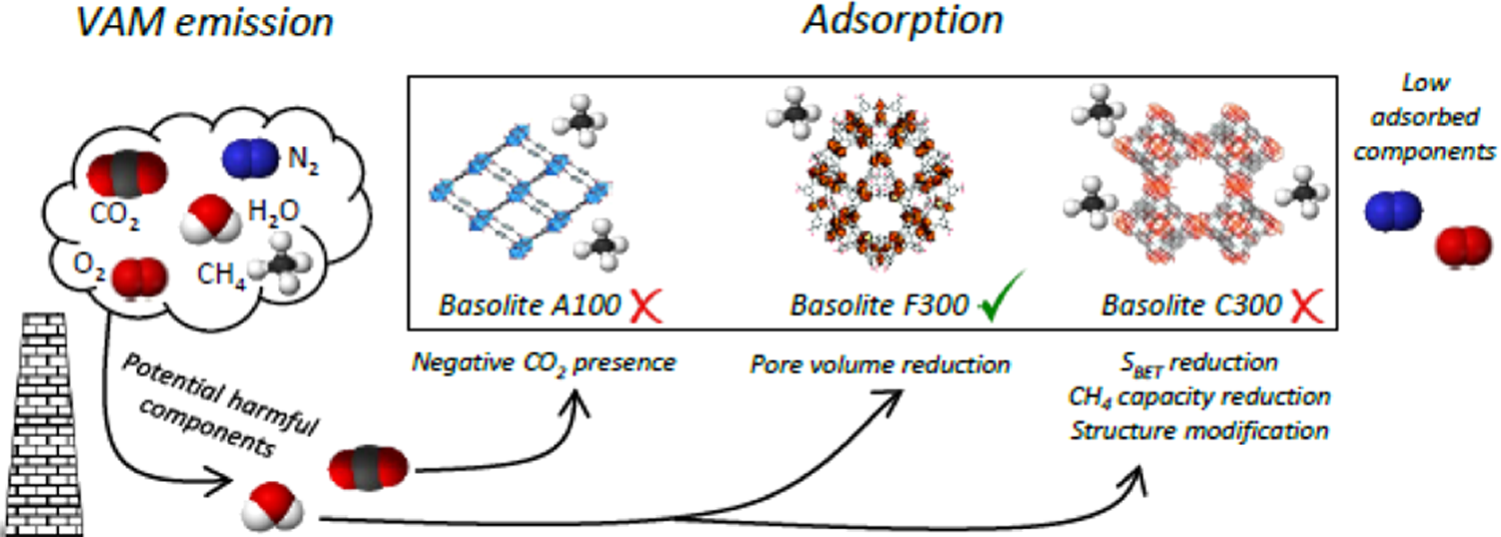

MOFs are potential adsorbents for methane separation from nitrogen, including recovery in diluted streams. However, water and carbon dioxide can seriously affect the adsorption performance. Three commercial MOFs, basolite C300, F300, and A100, were studied under similar conditions to fugitive methane streams, such as water (75 and 100% relative humidity) and carbon dioxide (0.33%) presence in a fixed bed. The presence of available open metal sites of copper (Cu^2+^) and aluminum (Al^3+^) in the case of basolite C300 and A100, respectively, constitutes a clear drawback under humid conditions, since water adsorbs on them, leading to significant methane capacity losses. Surprisingly, basolite F300 is the most resistant material due to its amorphous structure, which hinders water access. The combination of carbon dioxide and water creates a synergy that seriously affects basolite A100, closely related to its breathing effect, but does not constitute an important issue for basolite C300 and F300.

## 1 Introduction

Methane coal mining fugitive emissions constitute an important greenhouse gas (GHG) source but also a potential resource for both energy and chemicals. These fugitive streams are classified into coal bed methane (CBM), abandoned mine methane (AMM), and ventilation air methane (VAM).^1^ These streams are usually composed of methane, air, high relative humidity (100%), and traces of carbon dioxide (0.1%).^2^ The interest is mainly focused on obtaining energy directly through combustion in case of high methane concentrations (CBM and AMM, >30%), or just avoiding the direct emission to the atmosphere in case of low methane concentrations (VAM, <5%). However, VAM methane recovery and concentration for subsequent chemical upgrading or a more efficient thermal harnessing could be of interest. Swing adsorption techniques are established as the best available processes for methane recovery from diluted streams.^3^ Concerning suitable adsorbent materials, metal–organic frameworks (MOFs) have emerged as an alternative to activated carbons and zeolites because of their improved performance.^4^ In addition to the proven gas storage ability of these materials,^5^ MOFs were also widely studied in gas separations, such as methane from low-grade streams.^6,7^

The presence of other spectator species, such as moisture, can damage the adsorbent by either decreasing its adsorption capacity or by inducing serious structural modifications.^8^ In this way, Burtch et al.^9^ have reported a review correlating MOF structure with its sensitivity to water. More specifically, Canivet et al.^10^ have made a compilation of different water-sensitive MOFs, whereas Safy et al.^11^ have even developed a model to predict the harmful effect of moisture on different MOFs, observing a dramatic noxious effect for most. However, there are no studies on the effect of water on the structure or performance of commercial MOFs. Furthermore, the moisture effect on CO_2_ adsorption is widely studied in the literature,^12−14^ although studies about the humidity effect on methane adsorption are very scarce, especially for diluted streams.^15^ It should be noted that adsorption mechanisms can be very different for CO_2_ and CH_4_, leading to a different humidity effect on its adsorption.

Hence, this work studies the adsorption performance of three commercial MOFs, basolite C300, basolite F300, and basolite A100 at similar conditions to fugitive methane streams. These commercial materials are selected since they are those that have a synthesis process on an industrial scale, unlike most MOFs. The results, in addition to the adsorbent characterization, will provide information about the required features of the materials to be used in these recovery processes and the potential of the already available materials.

## 2 Materials and Methods

### 2.1 Materials

Three commercial materials supplied by BASF (basolite C300, C_18_H_6_Cu_3_O_12_, basolite F300, C_9_H_3_FeO_6_, and basolite A100, C_8_H_5_AlO_5_) were tested (96%, mass purity) in their original powder form. Gases were supplied by Air Liquide (>99.995% vol).

### 2.2 Adsorption Apparatus and Experimental Procedure

Fixed-bed adsorption studies were carried out in a stainless steel tube, 45 and 0.65 cm in length and internal diameter, respectively, filled with 0.15 g. Similar densities, around 0.35 g/cm^3^, make the bed lengths similar for each of the three materials. The fixed bed was operated in a tubular electric furnace (Nabertherm). In the adsorption stage, gas flows of air (47.5 mL/min) and methane (2.5 mL/min) were introduced by mass flow controllers (MFCs) previously calibrated (Bronkhorst), while the temperature was maintained at 298 K. In the desorption stage, 47.5 mL/min of air was introduced in the fixed bed, and the temperature was increased up to 423 K. The outlet of the fixed bed was analyzed by a mass spectrometer (Omnistar). A detailed scheme of the fixed-bed device is attached in Figure S1.

For experiments involving humidity, liquid water was introduced prior to the fixed bed, using a 5 mL liquid syringe (Hamilton) powered by a syringe pump (kdScientific). Water was immediately vaporized due to isolated heaters along the conductions, at 383 K. Water flow rates were selected based on the desired relative humidity (RH): 75 and 100%. Further, the materials were aged under a wet gas flow (100% RH) in the same fixed-bed device for 24 h prior to subsequent characterization. All of the fixed-bed adsorption experiments were conducted for three consecutive cycles. Further, all of the experiments were duplicated with deviations lower than 1% in all cases.

In addition, pure methane, nitrogen, and carbon dioxide adsorption were assessed using a thermal gravimetric analyzer (Setaram). Samples (15–30 mg) were pretreated at 423 K and 0.1 MPa under 60 mL/min of pure nitrogen for 2 h. Then, the measurement of mass changes under 60 mL/min of the desired gas was done at 298 K. All weight changes with respect to adsorption data were corrected using a blank calibration. The purge gas was nitrogen (40 mL/min).

### 2.3 Material Characterization

The morphology of the adsorbents, specific surface area, and pore volume were estimated by nitrogen physisorption at 77 K in ASAP 2020 (Micromeritics). Physisorption data were processed by using the Brunauer–Emmett–Teller (BET) model to determine the specific surface area of the materials. It was calculated in a range of *P*/*P*_0_ between 0.05 and 0.3, with correlation coefficients (*R*^2^) higher than 0.996 in all cases. Mesoporous volumes were estimated by the Barrett–Joyner–Halenda (BJH) method, whereas microporous volume was calculated using the Dubinin–Radushkevich method. Infrared spectra were acquired by DRIFT spectroscopy by a Thermo Nicolet FT-IR instrument (Nexus) equipped with an MCT/A detector. The sample of adsorbent (20 mg) was placed inside the temperature-controlled chamber. The material was pretreated with a mixture of methane (5%) and air (95%) with different RH (75 and 100%) at 298 K, followed by cleaning of the surface (423 K, helium) and a reflectance measurement of the passage of a dry-methane flowing mixture. All of the streams were 40 mL/min in total. The spectra were recorded in the 650–4000 cm^–1^ wavenumber range, subtracting the correspondent KBr standard background.

Crystallographic structures were determined by powder X-ray diffraction (PXRD) using a Philips PW 1710 diffractometer (Koninklijke Philips). The diffractometer works with the Cu–K_α_ line (λ = 0.154 nm) in the 2θ range of 5–85° at a scanning rate of 2°/min. Finally, SEM images were taken with a JEOL 6610LV (JEOL) scanning electron microscope.

## 3 Results and Discussion

### 3.1 Adsorption in Absence of Water and CO_2_

**Figure 1:**
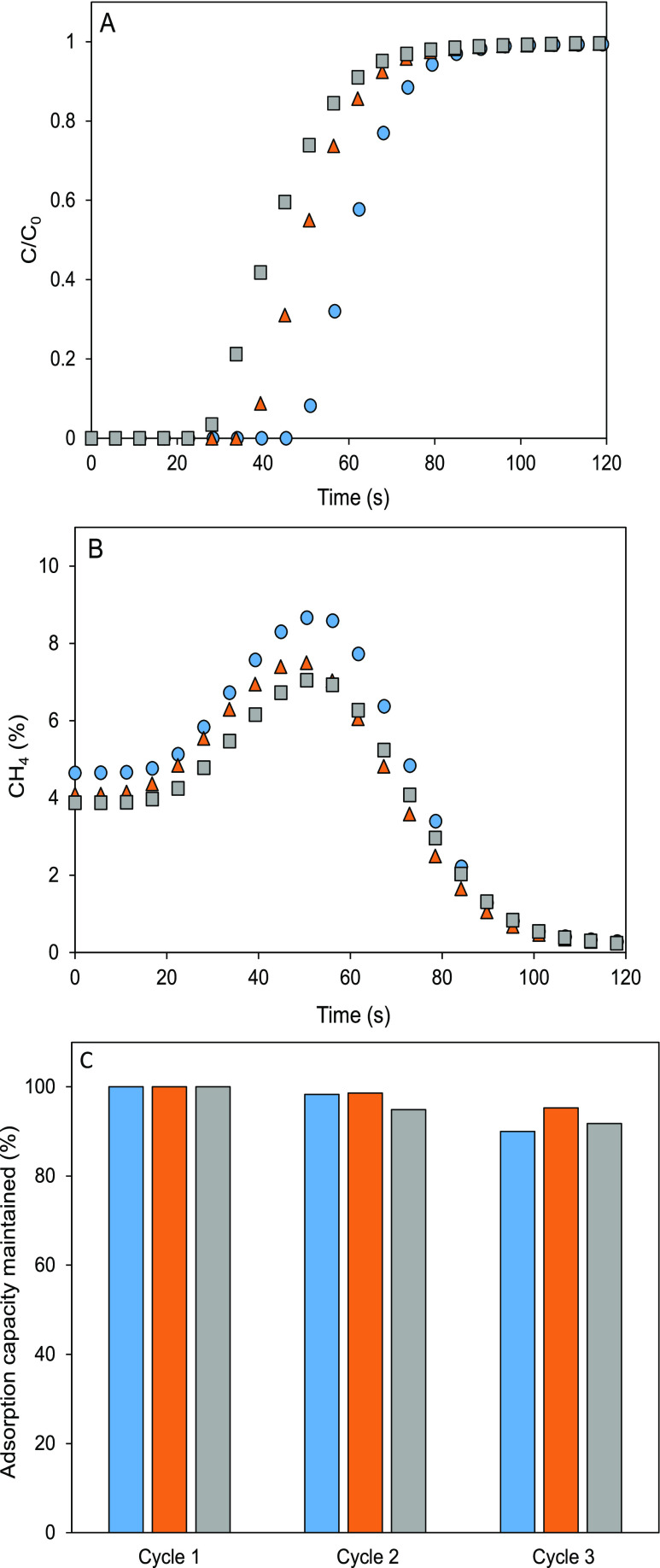
Adsorption (A) and desorption (B) curves for methane (*C*_0_ = 5%) and air (95%) in a fixed-bed device at 298 K in adsorption and 423 K in desorption (100% air). Methane adsorption capacity maintained after three consecutive cycles (C). Basolite C300 (blue), basolite F300 (orange), and basolite A100 (gray).

A simulated underground mining lean-methane emission with 5% CH_4_ and 95% air was used to test three different adsorbents in a fixed bed under mild conditions (298 K). Basolite C300 showed the highest methane adsorption capacity, 18.6 and 20.4% higher than those of basolite F300 and A100, respectively (Figure 1A). This trend correlates with the BET specific surface areas of the order of 1514 (C300) > 962 (F300) > 662 (A100) m^2^/g. The different adsorption behavior can be also justified by the structural differences among the materials.^16^ Basolite C300, homologous to HKUST-1, has copper ions with high affinity to methane.^17^ Basolite F300, with a distorted MIL-100(Fe) structure, has lower crystallinity and lower concentration of iron ions,^18^ with adsorbed molecules homogeneously distributed on the surface and not in specific areas.^19^ Basolite A100, homologous to MIL-53(Al), has a lower affinity to methane. It presents Al^3+^ open metal sites (OMS) available in the structure but with lower affinity to methane than in the case of copper ones.^19^

Desorption was performed after complete saturation. Figure 1B shows a maximum methane concentration increment after desorption of 41.7, 36.6, and 34.8% for basolite C300, F300, and A100, respectively, in comparison to the original feed stream (*C*_0_ = 5%). The higher increase in methane concentration observed in basolite C300 could tentatively be attributed to its higher methane heat of adsorption, as reported in a previous study.^20^ This may also justify the higher methane adsorption capacity compared to that of air constituents, as observed by thermogravimetry (Figure S2). Total adsorption capacities and selectivities are summarized in Table 1. Basolite A100 exhibits the best affinity toward methane but has the lowest adsorption capacities. Basolite C300 shows remarkable CH_4_/O_2_ separation capacity with the highest adsorption capacity but fails in CH_4_/N_2_ separation. Basolite F300 has moderate CH_4_/N_2_ separation capacity and low selectivity toward methane under oxygen presence.

**Table 1:** Physical Properties and Total Adsorption Capacities, Determined by Thermogravimetry (298 K, 0.1 MPa) at Pure Conditions and the Corresponding Selectivities with Respect to Methane

material	specific surface (m^2^/g)	pore (micro + meso) volume (cm^3^/g)	CH_4_ (mg/g)	N_2_ (mg/g)	O_2_ (mg/g)	CH_4_/N_2_	CH_4_/O_2_
basolite C300	1514	0.70 + 0.53	45.3	30.2	19.3	1.51	2.34
basolite F300	962	0.27 + 0.15	28.1	18.2	26.4	1.54	1.06
basolite A100	662	0.28 + 0.77	14.2	7.20	8.90	1.97	1.59

Adsorption capacity cycles (Figure 1C) show good stability with minimal loss of adsorption capacity, especially for basolite F300. These results show adequate adsorption capacity resistance in fixed-bed adsorption in the absence of humidity. Further, compared with other adsorbents in literature,^21,22^ materials studied in this work demonstrate competitive methane adsorption capacity and selectivity toward methane (Table 2). For example, they present similar or even higher methane adsorption capacities than other MOFs typically used for adsorption and separation, such as Al-CDC (20.96 mg/g), MOF-177 (8.18 mg/g), Ni-MOF-74 (22.75 mg/g), and HKUST-1 (13.15 mg/g), all of them measured at 298 K and 1 bar.^7^

**Table 2:** Total Adsorption Capacities Determined by Thermogravimetry (298 K, 0.1 MPa) for the Considered Materials of Carbon Dioxide at Pure Conditions and the Corresponding Selectivities with Respect to Methane

material	CO_2_ (mg/g)	CH_4_/CO_2_
basolite C300	117.5	0.38
basolite F300	48.8	0.57
basolite A100	58.3	0.24

### 3.2 Adsorption in the Presence of CO_2_

**Figure 2:**
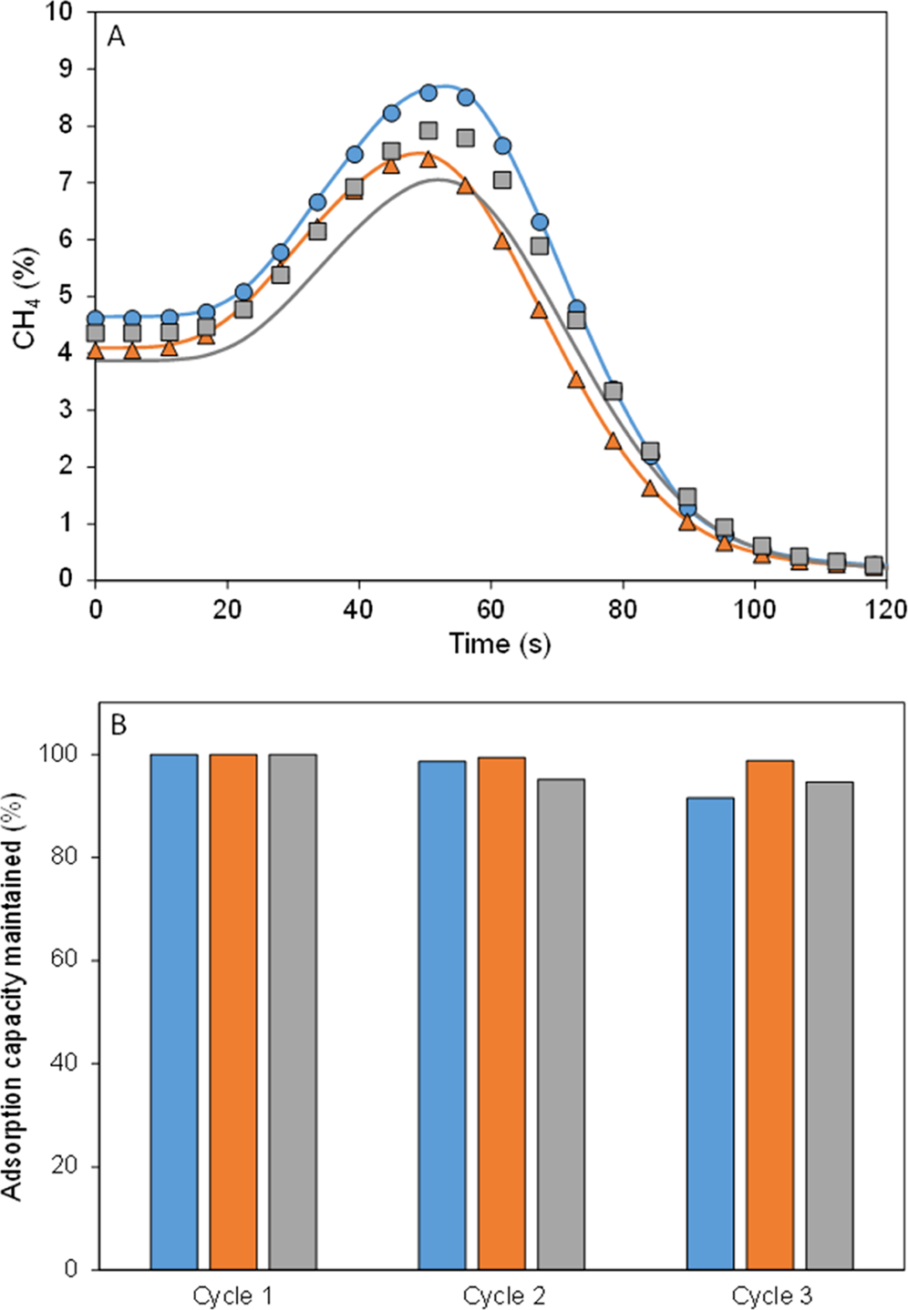
(A) Comparative of desorption curves of the three materials after methane (5%) and air (95%) adsorption (continuous line) and after methane (5%), air (balance), and carbon dioxide (0.33%) adsorption (pointed lines). (B) Methane adsorption capacity was maintained after three consecutive cycles. Basolite C300 (blue), F300 (orange), and A100 (gray).

The small size (3.33 Å) and high polarizability volume (2.51 Å^3^) of carbon dioxide could interfere with methane adsorption on MOFs.^7^ Therefore, the adsorption behavior of these materials in a 0.33% CO_2_, 5% CH_4_, and 95% air stream was studied in a fixed-bed device. After saturation, desorption was carried out in a manner analogous to that in the previous case (423 K, air). From Figure 2A, slight reductions in methane adsorption capacity are observed for basolite C300 (0.9%) and F300 (1.1%), with even an increase of 12.4% for A100. The variation of basolite A100, which can be considered significant, can be attributed to its long pore (lp) state,^23^ which facilitates the access to active metal centers by methane.^24^ Additionally, the selectivity of basolite A100 toward carbon dioxide is not too high, and the concentration is low, leading to an increase in methane adsorption capacity.

Three consecutive cycles were conducted in the presence of carbon dioxide (Figure 2B), and results show that carbon dioxide does not significantly affect the performance in consecutive cycles, with capacity losses similar to those observed for methane–air mixtures. Most materials used for methane and carbon dioxide separation in literature have a higher affinity toward carbon dioxide, as they are designed for biogas purification. Examples include porphyrin-based nanoporous organic polymers (PNOPs)^25^ and zeolitic-imidazolate framework (ZIF),^26^ with high carbon dioxide capacities and good CO_2_/CH_4_ selectivity, although low methane adsorption capacity. However, in the case of VAM, the carbon dioxide concentration is so low that it has practically no effect on performance. On the other hand, these considered materials may be suitable for the purification of biogas with representative amounts of CO_2_, as their adsorption capacity and selectivity are high.

Thermogravimetric results agree completely with fixed-bed curves (Table 2 and Figure S3), showing a poor performance for methane separation in the presence of carbon dioxide for basolite A100, whereas the methane adsorption capacities of basolite C300 and F300 are barely affected by the presence of carbon dioxide at very low partial pressures (0.33%). In fact, Teo et al.^27^ have demonstrated, by Monte Carlo simulations, that at carbon dioxide low partial pressure, it does not share adsorption sites with methane, so no carbon dioxide interference in methane adsorption occurs. On the other hand, in the case of basolite F300, Xian et al.^28^ have demonstrated, for MIL-100(Fe), a high influence of carbon dioxide concentration in the selectivity, so at such low carbon dioxide concentrations, the carbon dioxide does not affect the methane adsorption capacity.

### 3.3 Effect of Water on Methane Adsorption Behavior

**Figure 3:**
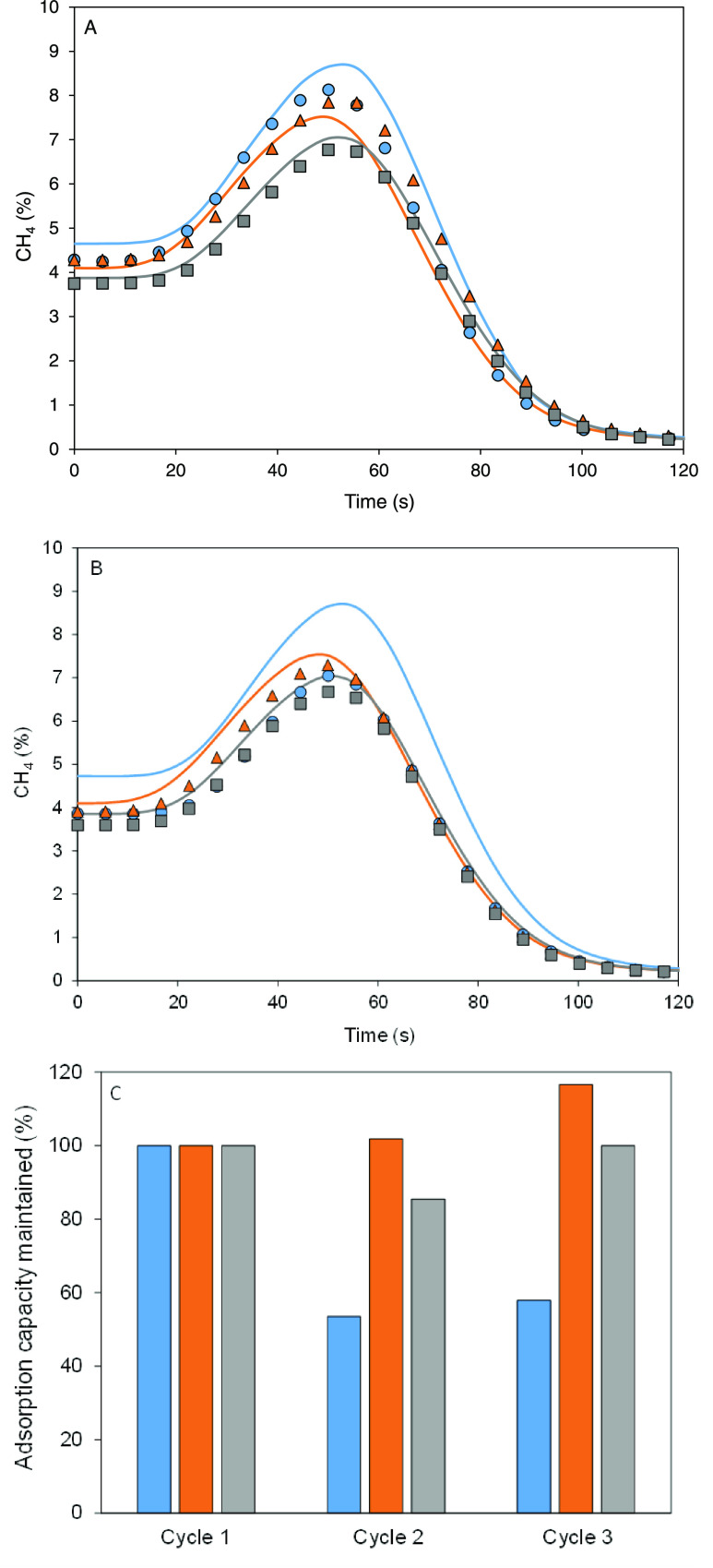
Comparison of desorption curves of the dry stream (continuous lines) and the same stream with different relative humidities (pointed lines): (A) 75% RH and (B) 100% RH. Methane adsorption capacity is maintained after three consecutive cycles at 100% RH (C). Basolite C300 (blue), basolite F300 (orange), and basolite A100 (gray).

The methane retention capacity of the three materials was tested at high relative humidities (RH) of 75 and 100%, which are representative of actual streams^29^ (Figure 3A,B). The lowest RH has little effect on the methane adsorption capacity, with basolite C300 showing the highest reduction, 6.2%. In fact, the relative humidity tested is high, and even other works have recorded decompositions of HKUST-1 from lower relative humidities.^30^ Water–MOF interactions and the presence of the OMS influenced the methane adsorption behavior. This interaction is weaker in the case of iron sites^31^ and hence humidity has a positive effect (4.6%) on the methane adsorption behavior of the Fe-containing MOF. This fact could be attributed to surface hydrates formed on the surface via hydrogen bonds, on which methane could be co-adsorbed due to its high polarizability.^32^ At the highest considered RH, basolite C300 is also the most affected material with a decrease of 18.6%, followed by basolite A100 (5.2%) and F300 (2.7%). The water pressure dependence of basolite F300 could be understood since the water that previously generated hydrates on the surface begins to cover the available surface and block these hydrophilic centers, causing finally a reduction in methane adsorption capacity.^32^ Further, experiments with three consecutive cycles (Figure 3C) showed that basolite C300 is the most adversely affected by water, with a decrease in capacity of 46.5% after the first cycle. Basolite F300 showed an increase in capacity after contact with water, while basolite A100 suffered a lower capacity loss (14.5%) compared to C300. These results highlight the significant impact of water on the adsorption performance.

Adsorption capacity, measured by thermogravimetry before and after water aging (Table 3), confirms that basolite C300 shows the highest loss in methane adsorption capacity due to water, indicating the significant influence of copper OMS in the adsorption mechanism, which becomes occupied by water. The CH_4_/N_2_ selectivity is particularly affected by the presence of water, decreasing from 1.51 to 1.18, due to the reduced dependence of N_2_ molecules on active metal sites for adsorption. Basolite F300 demonstrates the low influence of iron OMS on the adsorption mechanism, as the reduction in capacity is similar for both methane and nitrogen, likely due to minimal exposure of OMS compared to basolite C300, attributed to diffusion difficulties during the adsorption process.^33^ Basolite A100 shows a more pronounced reduction in methane adsorption capacity compared to nitrogen, indicating the influence of aluminum OMS. The CH_4_/N_2_ selectivity decreases from 1.9 to 1.7, suggesting the presence of specific areas, possibly near active metal centers, for methane adsorption.^19^

**Table 3:** Adsorption Capacity (298 K, 60 mL/min) Results for the Three Materials before and after the Water Treatment (100% RH, 24 h)

material	before/CH_4_ (mg/g)	before/N_2_ (mg/g)	after/CH_4_ (mg/g)	after/N_2_ (mg/g)
basolite C300	45.3	30.2	30.2	25.7
basolite F300	28.1	18.2	25.5	17.5
basolite A100	14.2	7.2	10.5	6.10

Limited research has been conducted on methane adsorption under humid conditions, with hydrophobic adsorbents being used in most cases. Some studies have shown that certain hydrophobic adsorbents,^34−36^ such as TUT-100 MOF and silicalite-1, maintain their selectivity and adsorption capacity for CH_4_/N_2_ separation even in humid environments. However, more experimentation is needed, as only a few adsorbents have been tested under these conditions.

Characterization of the materials after exposure to 100% RH shows a decrease in specific surface area and pore volume, indicating changes in the structure of the materials, especially for basolite C300 and A100 (Table 4 and Figure S4). Pristine samples exhibit a type II adsorption isotherm with a soft increase in the adsorbed volume in the range of 0.1–0.9 (*P*/*P*_0_), followed by steep adsorption with *P*/*P*_0_ greater than 0.95 for basolite A100 and, to a lower extent, in basolite C300. For basolite F300, an isotherm with similarities to type I is observed. Water induces changes in the isotherms, especially for C300 and A100, for which isotherm transitions to the patterns of type IV are shown. This isotherm is characteristic of mesoporous materials. Basolite A100 presents a barely distinguishable hysteresis loop in the range *P*/*P*_0_ 0.90–0.99, which is an indication of the interparticle porosity. Basolite C300 exhibits an H3-type hysteresis loop at *P*/*P*_0_ 0.42–0.97, representative of slitlike pores. Hence, water enhances adsorption in the mesoporous zone, with multilayer adsorption and condensation phenomena at high pressures in the detriment of adsorption in micropores. It is remarkable that basolite F300 experiments show enhanced adsorption due to mesopore contribution, justifying the limited water effect on methane adsorption on this material. In the case of basolite A100, the morphological changes are not directly correlated to the methane adsorption capacity, remaking in this way the influence of the aluminum OMS.

**Table 4:** Morphological Features of Each Material before and after Water Treatment (100% RH, 24 h)

material	pristine BET (m^2^/g)	pristine mesopore volume (cm^3^/g)	pristine micropore volume (cm^3^/g)	treated BET (m^2^/g)	treated mesopore volume (cm^3^/g)	treated micropore volume (cm^3^/g)
basolite C300	1515	0.53	0.70	695	0.75	0.18
basolite F300	962	0.15	0.27	697	0.24	0.08
basolite A100	656	0.77	0.28	307	0.68	0.14

DRIFT analyses reveal water-induced changes in the structure of basolite C300, whereas, for basolite F300 and A100, no structural change was observed after the water treatment (Figure S5). That figure includes spectra of the materials recorded at 298 K without contact with water, and after aging at RH of 75 and 100% directly on the equipment. In the case of basolite C300 spectra, clear bands characteristic of the material are observed below 2000 cm^–1^: the peaks in the range 1300–1500 and 1500–1700 cm^–1^ are related to −O–C–O– groups, whereas peaks in 1374–1559 cm^–1^ correspond to double bonds C=C, which demonstrate the incorporation of the organic ligand 1,3,5-BTC in the structure. Furthermore, the band in the region 3200–3500 cm^–1^ can be related to the −OH bond of water to the structure.^37^ As seen, a continuous displacement of bands at 1500–1700 cm^–1^ and the apparition of new peaks, corresponding to −O–C–O– groups, is observed with an increasing number of cycles, suggesting water-induced changes in the structure. Basolite F300 shows a similar spectrum to basolite C300 due to the presence of the same organic ligand. Additionally, there is no displacement or appearance of new peaks along all of the wavelength range, discarding structural changes due to water presence. In the case of basolite A100, peaks at 760 cm^–1^ correspond to the vibration of hydrogen in the aromatic ring, whereas peaks at 860 and 1025 cm^–1^ are related to the carboxyl bonds and between 1460 and 1700 cm^–1^ to the double bond C=C.^38^ Similarly to Basolite F300, any structural change is observed after the water treatment, without displacement or the appearance of new peaks. In addition, the three MOFs present an intense water desorption capacity at high a temperature (423 K), since the band corresponding to the −OH link (3200–3550 cm^–1^) is low in all of the three cycles for each material, which indicates that there is no water left after the cleaning stages. These bands are a little higher in the case of basolite C300, which presents more hydrophilic metal centers and desorbs water more slowly at the same temperature than the other two materials. In fact, these active centers are the ones that cause the greatest reduction in methane adsorption capacity in the presence of water for basolite C300.

Figure S6 presents PXRD diffractograms depicting moisture-induced crystallinity changes in three different scenarios. Basolite C300 shows changes in peak intensity at 6.7 and 11.6°, indicating changes in crystallinity but not in the crystalline structure.^30^ These changes can be related to the additional fissures and agglomeration observed by SEM (Figure S7), as well as the −O–C–O– group displacement observed by DRIFT. Basolite F300, which has a low-crystalline structure, does not exhibit significant changes due to the humidity. However, basolite A100 shows a significant increase in the amorphous phase, particularly at 2θ values higher than 15°, and the appearance of two new peaks around 25°, suggesting crystalline phase changes induced by water. This is similar to previous findings after pressurization,^39^ indicating phase changes and amorphization. These results suggest that methane adsorption is not strongly dependent on the crystal structure of the adsorbent material but rather on other morphological features such as specific surface area or total pore volume.

### 3.4 Adsorption at Actual Conditions

**Figure 4:**
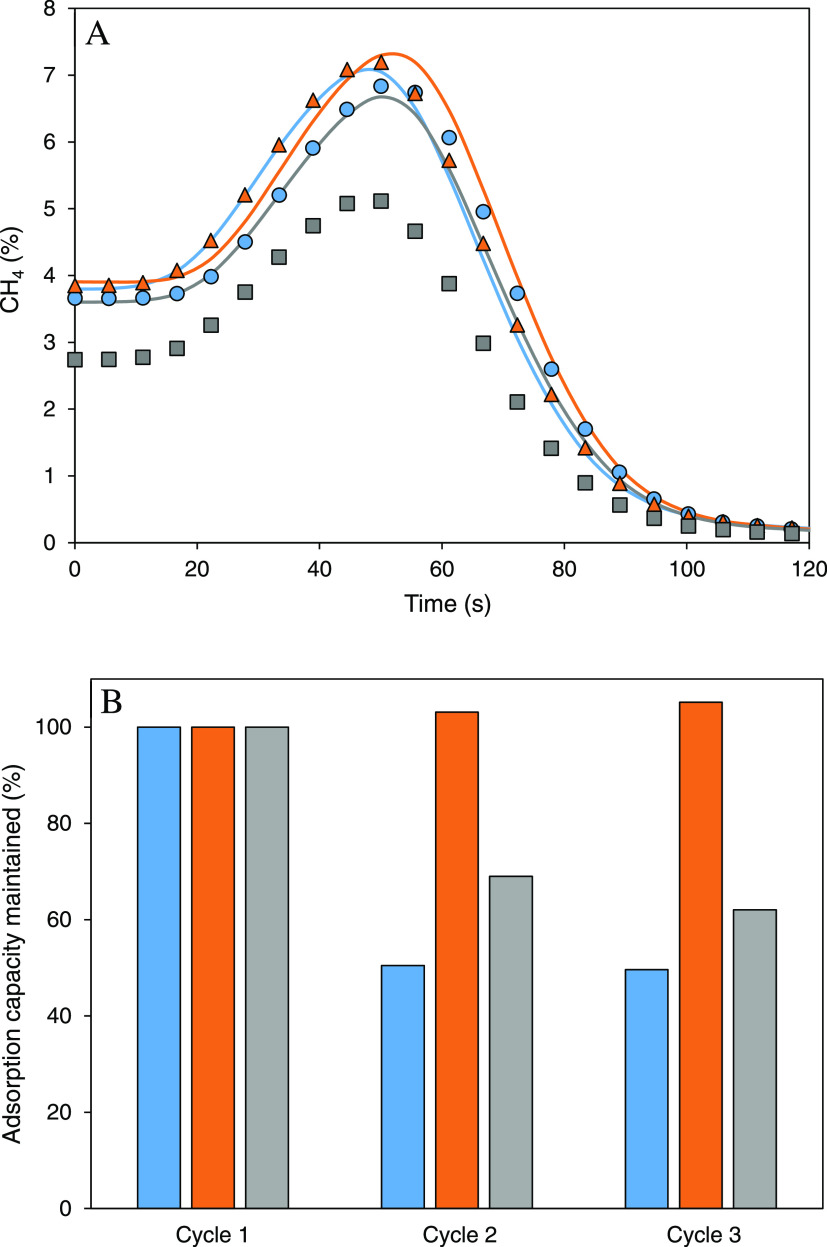
(A) Comparison of desorption curves for methane, air, and water (RH: 100%) (continuous line) and the same stream with carbon dioxide (0.33%) (pointed line). (B) Methane adsorption capacity maintained after three consecutive cycles of the last stream considered. Basolite C300 (blue), basolite F300 (orange), and basolite A100 (gray).

Figure 4A shows the effect of carbon dioxide (0.33%) in the case of wet streams (100% RH), reproducing similar conditions to an actual stream to be treated. Basolite C300 and F300 show minimal influence of CO_2_, with a slight reduction in methane adsorption capacity after simultaneous exposure to carbon dioxide and water (Table 5). This is consistent with previous works indicating that strong interaction between CO_2_ and H_2_O leads to increased CO_2_ adsorption capacity in the presence of water.^40^ However, basolite A100 exhibits detrimental effects with the simultaneous presence of carbon dioxide and water, causing modifications in the breathing structure and significantly reducing methane adsorption capacity.^41^

**Table 5:** Effect of H_2_O (100% RH, 24 h) and CO_2_ (0.33%) Presence in Total CH_4_ Adsorption Capacity in Comparison to the Dry Conditions Experiment

material	capacity reduction with H_2_O (%)	capacity reduction with CO_2_ (%)	capacity reduction with H_2_O and CO_2_ (%)
basolite C300	18.6	0.9	21.1
basolite F300	2.7	1.1	4.1
basolite A100	5.2	12.4*	18.1

* In the case of basolite A100, the CO_2_ presence provokes a methane adsorption capacity increase.

Three consecutive adsorption cycles in the presence of carbon dioxide and water (Figure 4B) show similar results for basolite C300 and F300 as in the case of methane, air, and water. However, basolite A100 shows a substantial reduction in methane adsorption capacity with cycles. To the best of our knowledge, there is no previous work that has studied the confluence of methane, air, carbon dioxide, and water in a dynamic adsorption study. Most literature suggests the dehumidification of streams prior to passing through fixed beds, but this would be costly for recovering low-concentration gaseous waste.^42^

## 4 Conclusions

The effect of water and carbon dioxide on the methane adsorption performance of three commercial MOFs (basolites C300, F300, and A100) is studied in this work. At the considered concentrations (0.33%), carbon dioxide has a limited impact on the methane adsorption capacity of C300 and F300 since there is no competition with methane for adsorption sites. On the other hand, A100 shows an increase in methane adsorption capacity due to its breathing effect, enabling methane penetration into the structure. The presence of water (75–100% RH) largely hinders methane adsorption, especially for basolite A100 and more markedly for C300. By contrast, basolite F300, with a distorted structure, prevents easy access of water to iron OMS, and even enhances methane adsorption, making it the most promising material for low-grade methane recovery in humid streams. Basolite F300 also shows the best behavior in the presence of water and CO_2_, suggesting its potential for large-scale methane separation processes at real conditions, and opening the possibility of using commercial MOFs in scalable processes after pilot plant experimentation.

## Supporting Information Available

Fixed-bed device scheme; methane, nitrogen, and oxygen adsorption curves determined by thermobalance; carbon dioxide adsorption curve obtained by thermobalance; nitrogen physisorption measurements of pristine and moist-treated materials; DRIFT obtained patterns for aged MOFs; PXRD obtained profiles before and after water treatment; and SEM images before and after water treatment (PDF)

## Abbreviations

MOFs: metal–organic frameworks
GHG: greenhouse gases
CBM: coal bed methane
AMM: abandoned mine methane
VAM: ventilation air methane
MFC: mass flow controller
RH: relative humidity
DRIFT: diffuse reflectance for infrared Fourier transform spectroscopy
FT-IR: Fourier transform infrared reflectance
PXRD: powder X-ray diffraction
SEM: scanning electron microscopy
OMS: open metal sites
*C*_0_: initial methane concentration (mol/m^3^)
*C*: methane concentration (mol/m^3^)
lp: large-pore phase
PNOPs: porphyrin-based nanoporous organic polymers
ZIF: zeolitic-imidazolate framework
