# Supplementary material for: Effect of Water and Carbon Dioxide on the Performance of Basolite Metal–Organic Frameworks for Methane Adsorption
Source: Energy Fuels. 2023 Sep 27;37(19):14836–44. doi: 10.1021/acs.energyfuels.3c02393 (PMC10561151; doi:10.1021/acs.energyfuels.3c02393)
Supplement: Supplementary file 1 — ef3c02393_si_001.pdf [file ef3c02393_si_001.pdf]

Supporting Information

Effect of water and carbon dioxide on the performance of Basolite metal-organic frameworks for methane adsorption

David Ursueguía, Eva Díaz, Salvador Ordóñez*

[a]Catalysis, Reactors and Control Research Group (CRC), Department of Chemical and Environmental Engineering, University of Oviedo, Julián Clavería s/n, 33006 Oviedo, Spain

*e-mail: sordonez@uniovi.es, Tel: +34 985 103 437, Fax: +34 985 103 434

Table of Contents

Figure S1. Fixed-bed device scheme. S3

Figure S2. Adsorption of methane, nitrogen and oxygen at 298 K on the three pristine materials, determined by thermobalance. S4

Figure S3. Adsorption of carbon dioxide at 298 K on the three pristine materials determined by thermobalance. S5

Figure S4. Nitrogen physisorption measurements at 77 K and comparison between pristine materials and moist-treated materials. S6

Figure S5. DRIFT patterns of the MOFs aged in presence of different gaseous mixtures adsorption (5% CH4, 95% air, 298 K and 40 ml/min). S7

Figure S6. PXRD profiles of the three materials before and after water treatment (100% RH). S8

Figure S7. SEM images of the three materials before and after water treatment (100% RH, 24 h). S9

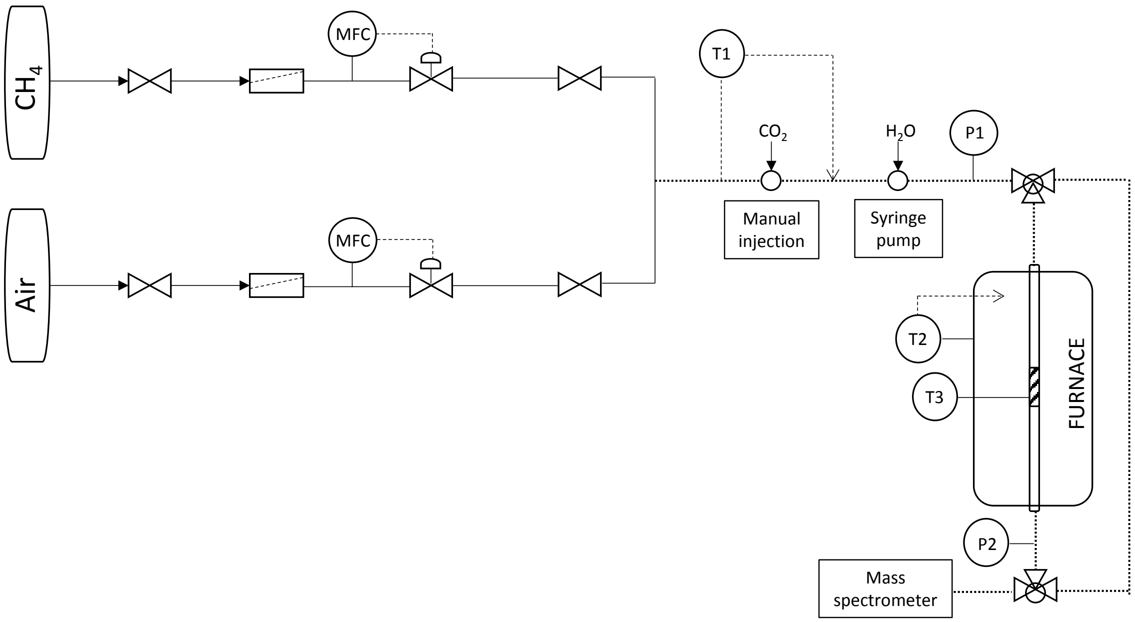

Figure S1. Fixed-bed device scheme. Dotted line points out the heated and isolated line. Discontinuous line indicates the electrical signals.

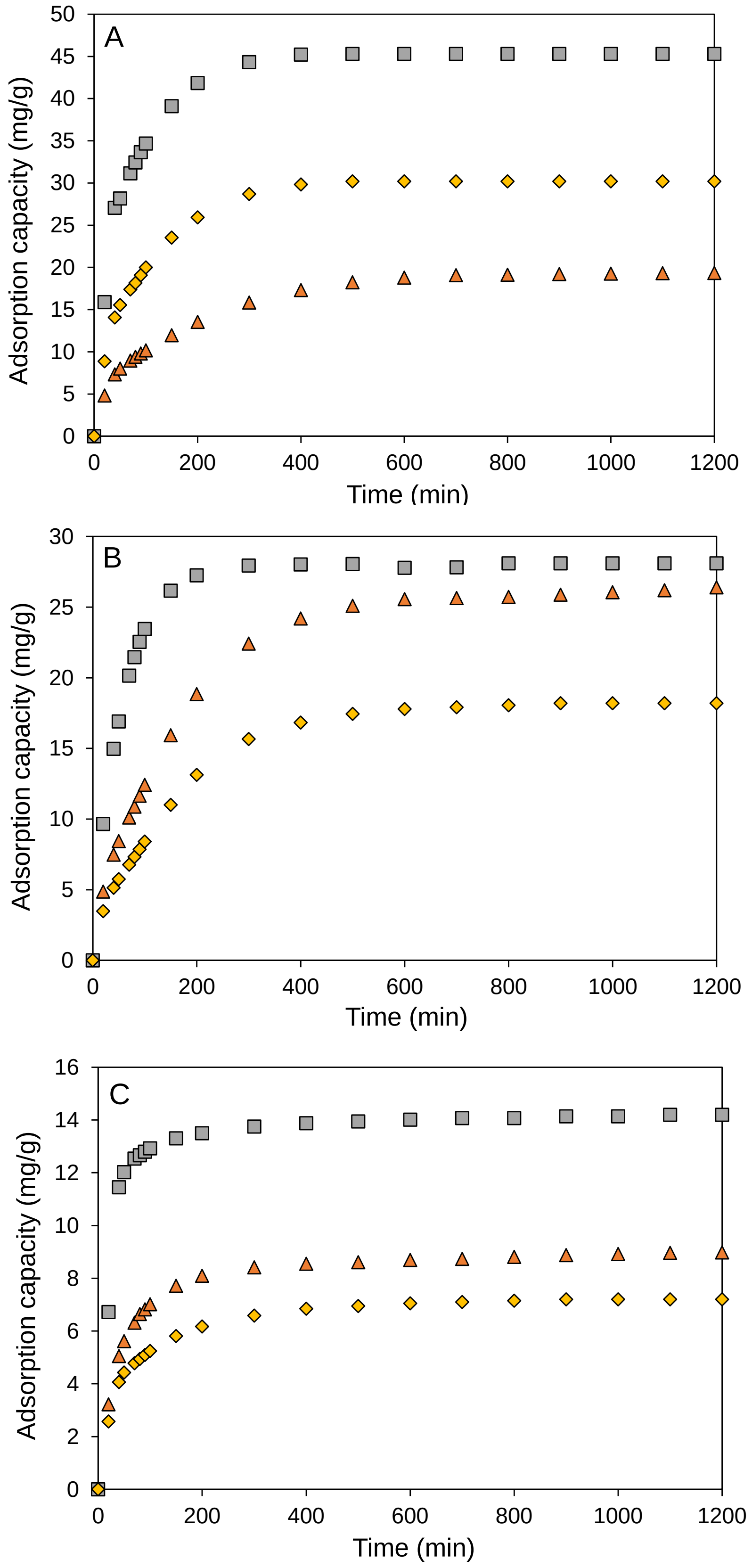

Figure S2. Adsorption of methane (grey squares), nitrogen (yellow rhombus) and oxygen (orange triangles) at 298 K on the three pristine materials, determined by thermobalance. Basolite C300 (A), Basolite F300 (B) and Basolite A100 (C).

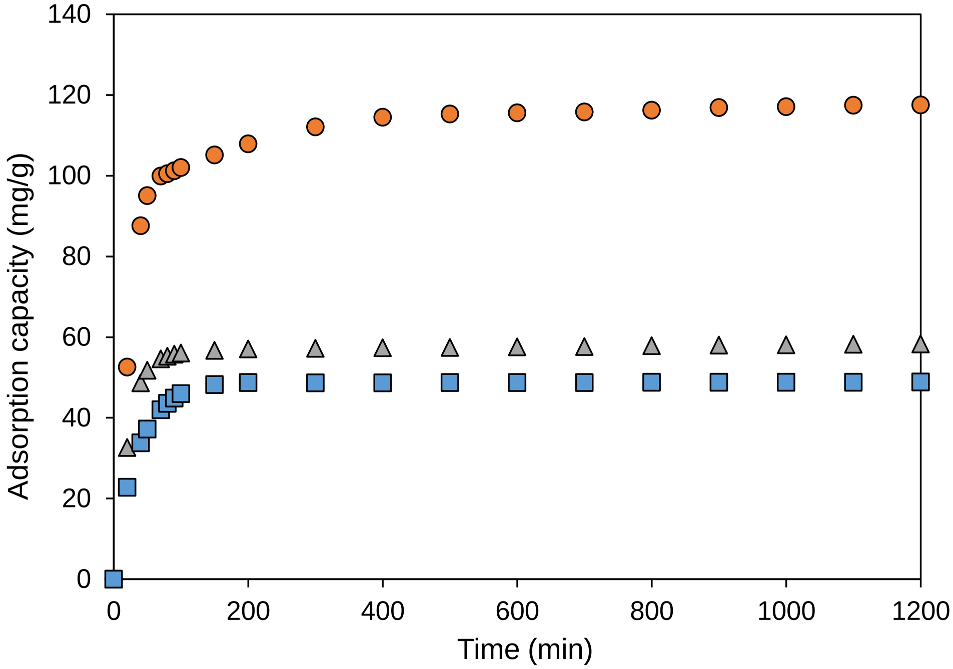

Figure S3. Adsorption of carbon dioxide at 298 K on the three pristine materials (orange circles: Basolite C300; grey triangles: Basolite A100; blue squares: Basolite F300), determined by thermobalance.

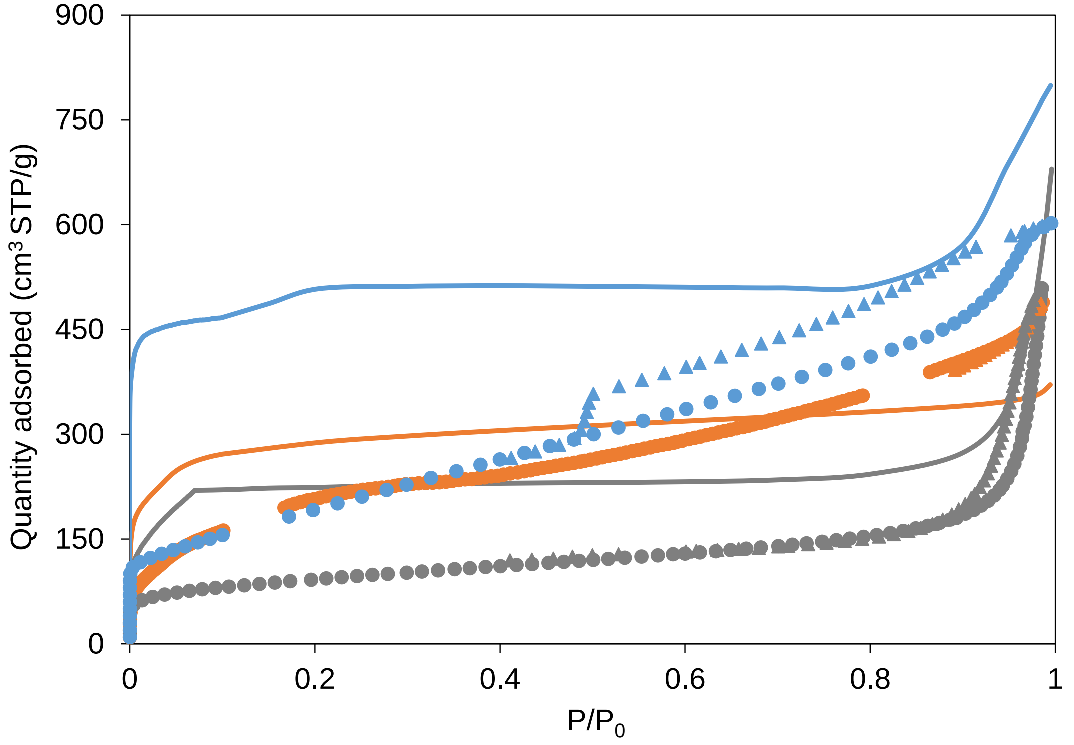

Figure S4. Nitrogen physisorption measurements at 77 K and comparison between pristine materials (solid line) and moist-treated materials (circles line for adsorption and triangles line for desorption). Basolite C300, blue; Basolite F300, orange and Basolite A100, grey.

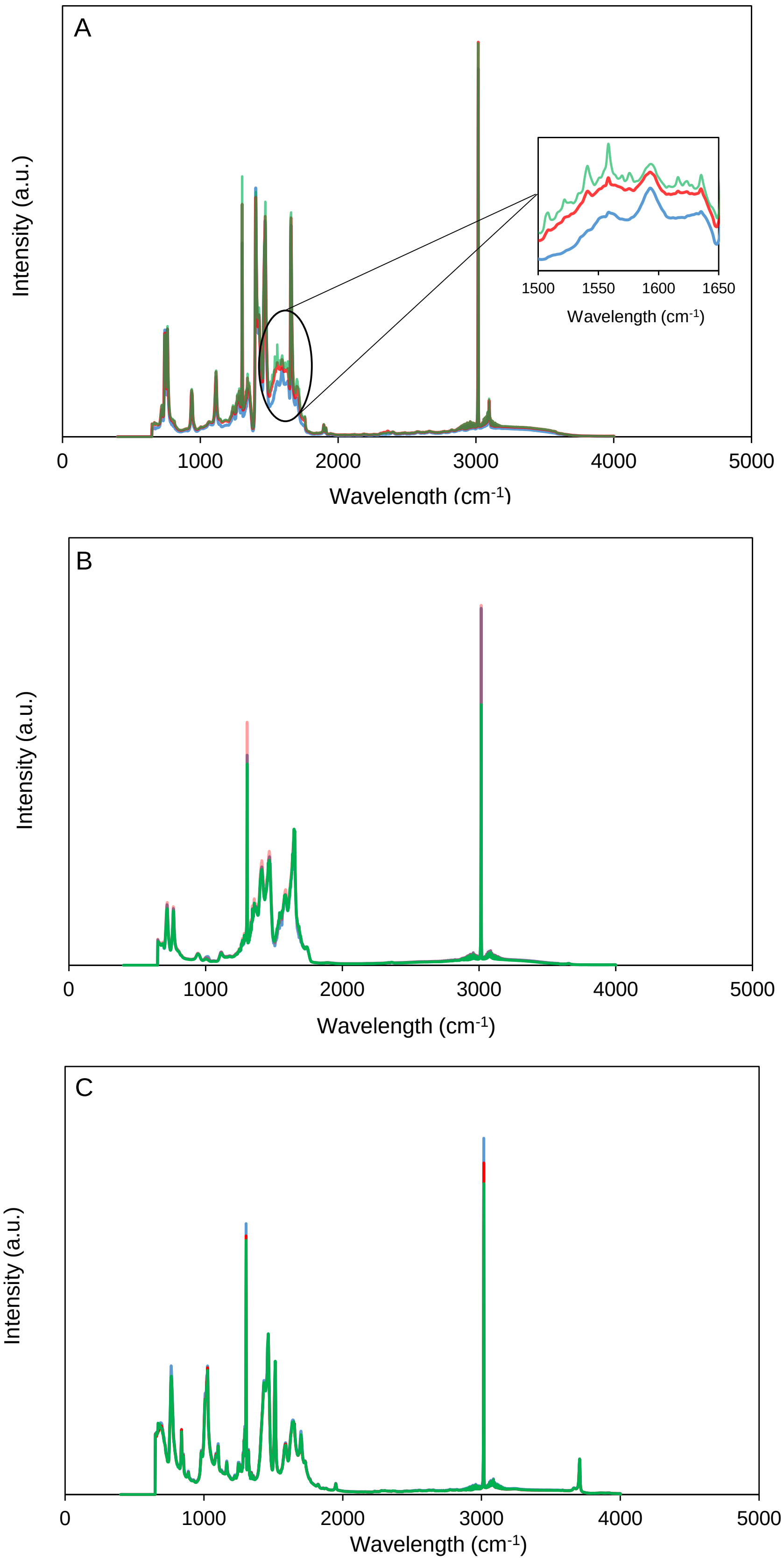

Figure S5. DRIFT patterns of the MOFs aged in presence of different gaseous mixtures adsorption (5% CH_4, 95% air, 298 K and 40 ml/min) (A: C300, B: F300, C: A100). 5% CH_4, 95% air, blue; 5% CH_4, 95% air, 75% RH, red; 5% CH_4, 95% air, 100% RH, green.

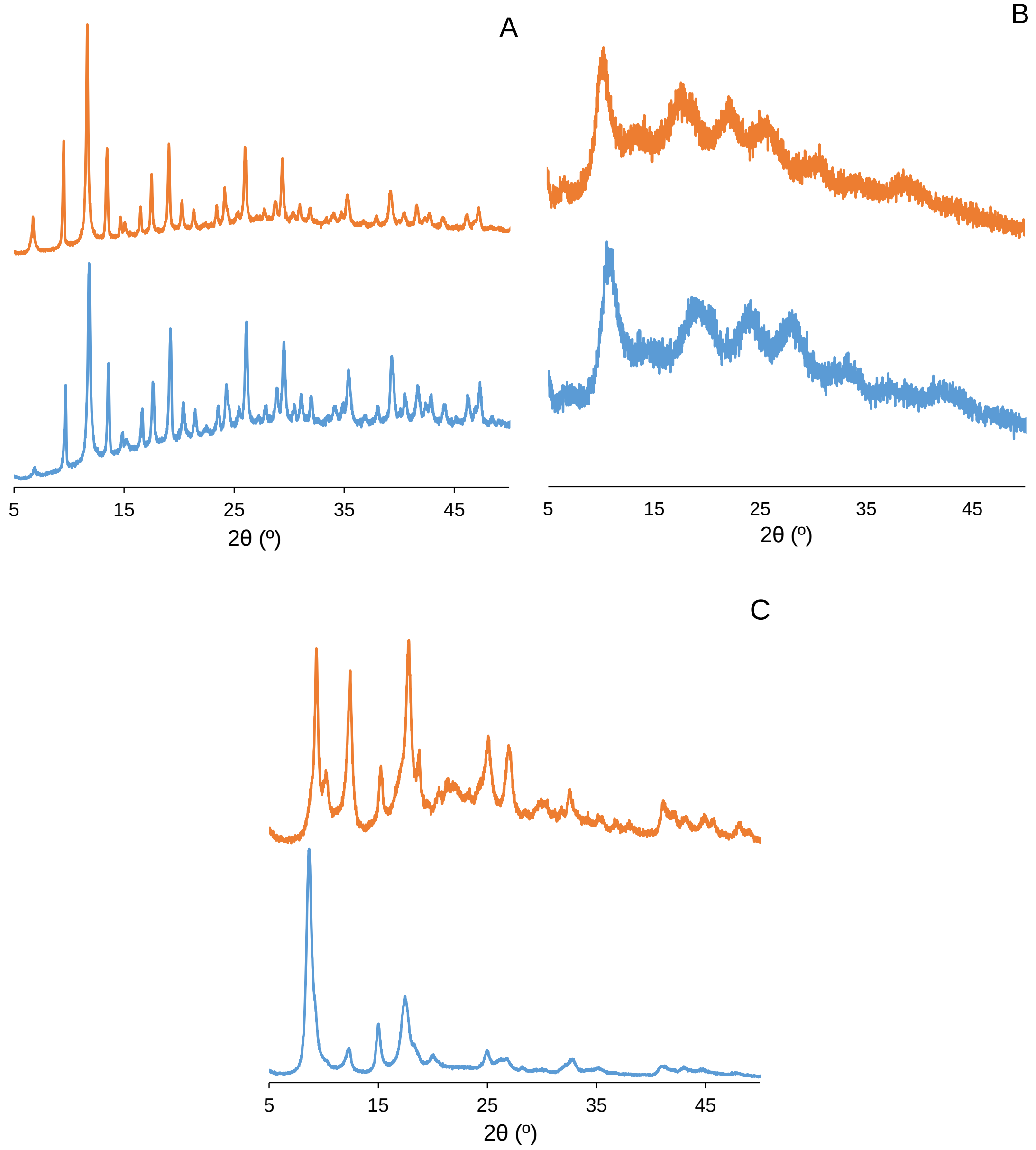

Figure S6. PXRD profiles of the three materials before (blue) and after (orange) water treatment (100% RH). Basolite C300 (A), Basolite F300 (B), Basolite A100 (C).

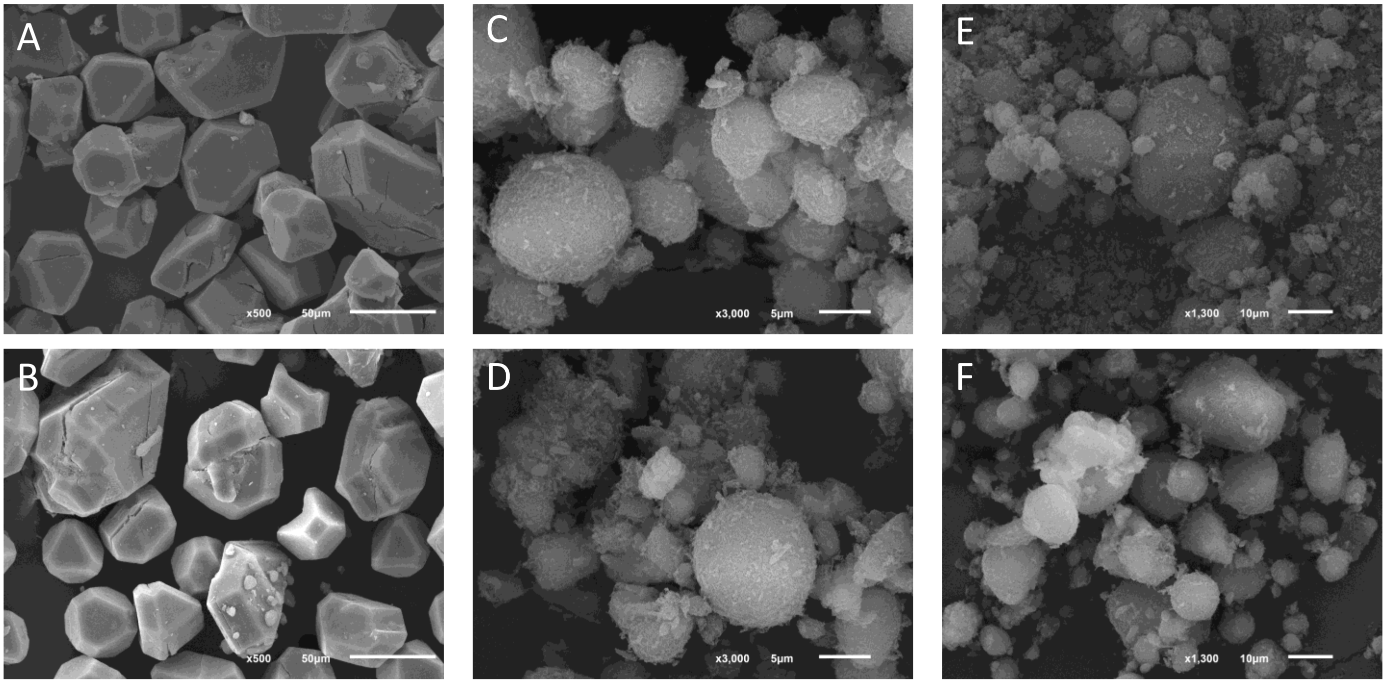

Figure S7. SEM images of the three materials before and after water treatment (100% RH, 24 h). Basolite C300 (A: Before, B: After), Basolite F300 (C: Before, D: After), Basolite A100 (E: Before, F: After). Scale and total magnification are reflected at the bottom of each photograph.
